# A simple device for assisting capsule endoscopy in passing through complex duodenal stenosis

**DOI:** 10.1055/a-2781-5874

**Published:** 2026-02-24

**Authors:** Pingping Zhang, Chaomei Lian, Zimao Jiang, Kaiming Wu, Peiqin Wang

**Affiliations:** 1 12520 Department of Gastroenterology, Changhai Hospital, Naval Military Medical University, Shanghai, China; 2 Department of Gastroenterology, Quanzhou Taiwan Investment Hospital, Quanzhou, China; 3 56652 Department of Gastroenterology, Changzheng Hospital, Naval Military Medical University, Shanghai, China

Retention of the capsule endoscope (Medtronic SB3, 11.4 × 26.2 mm) could lead to incomplete digestive tract examination and delayed diagnosis 1 . We report a method to assist capsule endoscopy in passing through the difficult-to-navigate duodenal bulb ( Video 1 ).

A simple device for assisting capsule endoscopy passage through complex duodenal stenosis.Video 1

**Fig. 1:**
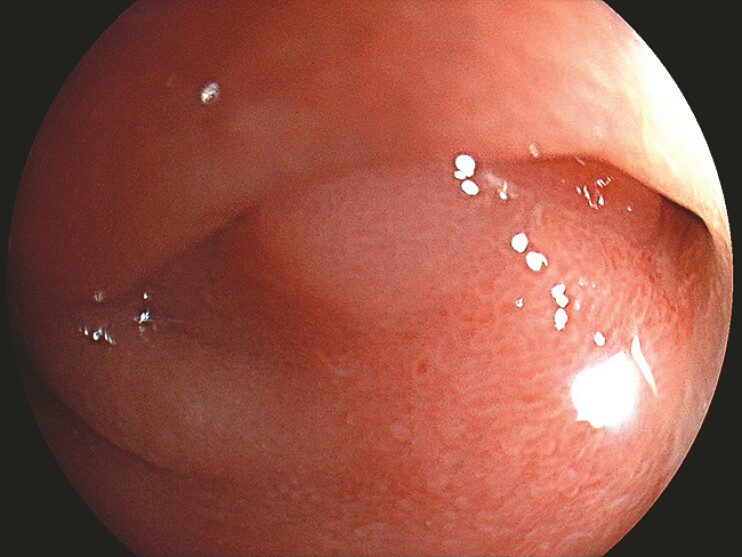
A stricture was revealed at the bulb–descending junction.

**Fig. 2:**
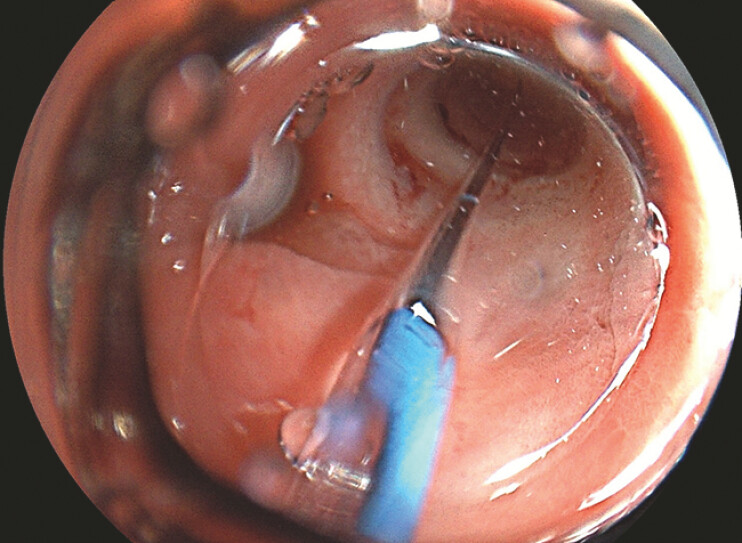
The stricture was dilated to 1.2 cm with the CRE balloon.

**Fig. 3:**
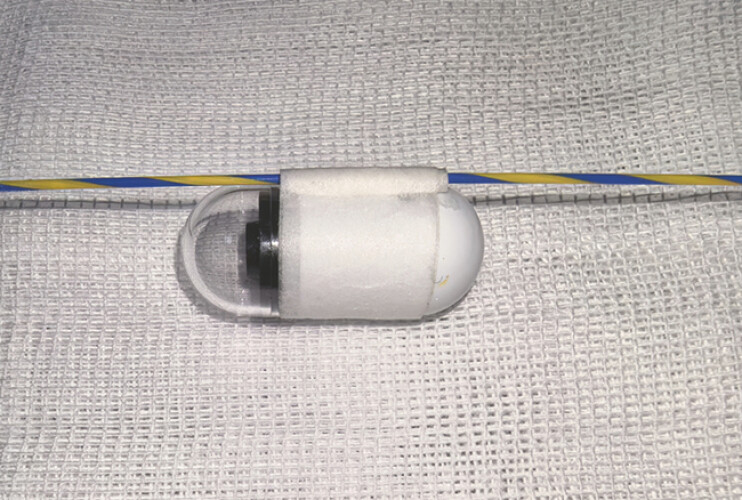
The guidewire passes through the guidewire channel fabricated from adhesive tape and secured to the capsule endoscope.

**Fig. 4:**
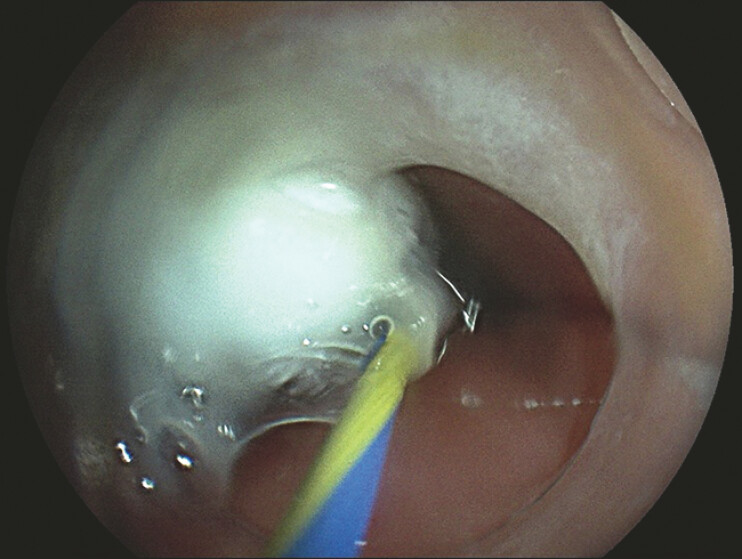
The capsule endoscope was inserted along the guidewire.

**Fig. 5:**
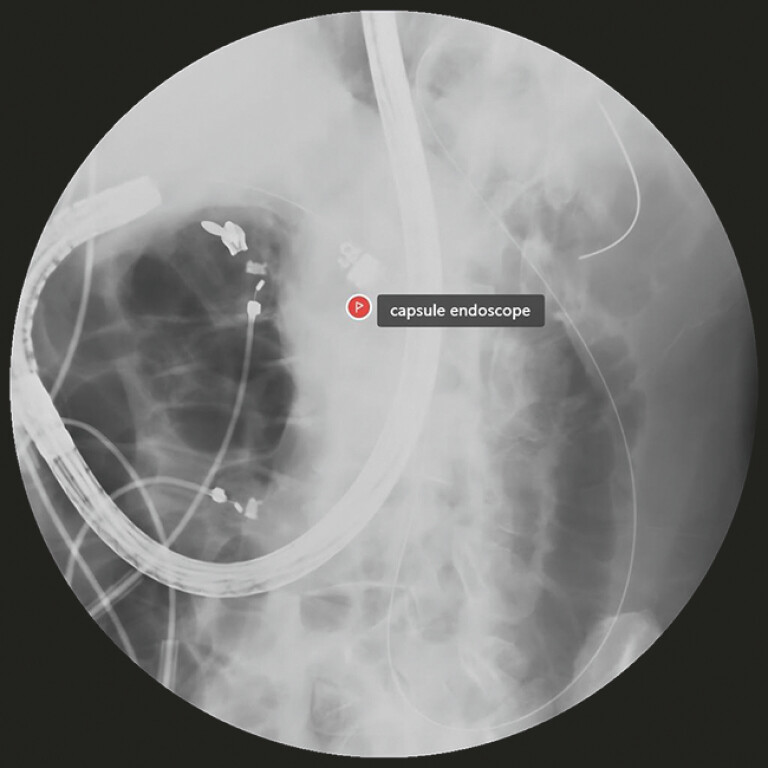
The capsule endoscope was successfully placed in the duodenum under X-ray surveillance.

A 58-year-old man presented with a history of duodenal bulb ulcer, and the patient was admitted to our hospital for capsule endoscopy examination due to gastrointestinal bleeding. During gastroscopy, the duodenal bulb mucosa was observed to be clustered, and a stricture was revealed at the bulb–descending junction with a diameter of approximately 0.7 cm ( Fig. 1 ), which could not be traversed via a conventional endoscope. A CRE balloon was inserted through the instrument channel and the stricture was dilated to 1.2 cm with significant resistance ( Fig. 2 ), so further dilation was not performed. The gastroscope (diameter: 0.99 cm) scraped through after the dilation, and it was anticipated that it would be difficult to place the capsule endoscope into the duodenum using conventional methods. Therefore, a small hollow tube was formed using medical pressure-sensitive (width: 1.25 cm and thickness: 0.1 mm) adhesive tape to serve as a guidewire channel ( Fig. 3 ), and the hollow tube was attached to the body of the capsule endoscope using the tape. Afterwards, a guidewire (0.035 inch) was placed into the duodenum through the instrument channel, and the capsule endoscope was inserted along the guidewire ( Fig. 4 ). With the assistance of the endoscope tip pushing, the capsule endoscope was successfully placed in the duodenum under X-ray surveillance ( Fig. 5 ).

Currently, common methods to address capsule endoscope retention include snares, retrieval baskets, specialized delivery devices, and prokinetic agents 2 . In this study, we reported a case where a simple guidewire channel made of adhesive tape was attached to the body of the capsule endoscope, allowing the capsule to guide the descending duodenum along the guidewire. This method could provide additional options for capsule retention in clinical practice.

Endoscopy_UCTN_Code_TTT_1AP_2AB
